# Being Sticker Rich: Numerical Context Influences Children’s Sharing Behavior

**DOI:** 10.1371/journal.pone.0138928

**Published:** 2015-11-04

**Authors:** Tasha Posid, Allyse Fazio, Sara Cordes

**Affiliations:** 1 Department of Psychology, The Ohio State University, Columbus, Ohio, United States of America; 2 Department of Psychology, Boston College, Chestnut Hill, Massachusetts, United States of America; UNC School of Dentistry, University of North Carolina-Chapel Hill, UNITED STATES

## Abstract

Young children spontaneously share resources with anonymous recipients, but little is known about the specific circumstances that promote or hinder these prosocial tendencies. Children (ages 3–11) received a small (12) or large (30) number of stickers, and were then given the opportunity to share their windfall with either one or multiple anonymous recipients (Dictator Game). Whether a child chose to share or not varied as a function of age, but was uninfluenced by numerical context. Moreover, children’s giving was consistent with a proportion-based account, such that children typically donated a similar proportion (but different absolute number) of the resources given to them, regardless of whether they originally received a small or large windfall. The proportion of resources donated, however, did vary based on the number of recipients with whom they were allowed to share, such that on average, children shared more when there were more recipients available, particularly when they had more resources, suggesting they take others into consideration when making prosocial decisions. Finally, results indicated that a child’s gender also predicted sharing behavior, with males generally sharing more resources than females. Together, findings suggest that the numerical contexts under which children are asked to share, as well as the quantity of resources that they have to share, may interact to promote (or hinder) altruistic behaviors throughout childhood.

## Introduction

Adults promote the social norm of sharing and consider altruism a positive cooperative behavior (Nowak, 2006); however, only recently have investigations begun to explore whether infants and young children feel and act similarly [1,2,3]. While evidence points to the existence of an “early moral compass,” with children spontaneously sharing resources with anonymous recipients even when not expected to do so [1,4], little is known regarding the limits or conditions of these altruistic tendencies (e.g., [5]). Studies using a variety of strategic and economic games have shed light on the circumstances that foster or hinder altruistic behaviors (i.e., giving, sharing) in adults, determining when a preference for equity occurs [6,7,8,9]. In the same vein, the current study explores the circumstances contributing to the exhibition of altruistic behaviors in children by manipulating the availability of resources and the number of potential recipients that a child encounters. In doing so, this investigation explores children’s decision-making related to unsolicited resource distribution across development, as well as what contexts may promote or hinder these prosocial tendencies.

### Background

Much research on altruistic behavior has involved an anonymous first-person, economic paradigm called the Dictator Game (“DG,” [10]). In the standard DG, one person (the dictator) is given a set amount of resources (e.g., money) and then is asked to decide how much of the resource to keep for him- or herself and how much to give away to an anonymous source (the receiver). In this task, expectations of reciprocity (e.g., “If I am nice to this person, then they will be nice back to me”) should not motivate the dictator’s decisions since the dictator and the receiver are anonymous to one another. Economically speaking, the most rational decision would be to not leave anything for the receiver. However, most adults do donate some of the resource, giving approximately 20–30% to an anonymous recipient, with modal offers varying from 0% to 50% [8,10].

Developmental investigations involving the DG have also revealed spontaneous sharing behavior in children as early as preschool-age. In these tasks, children are provided stickers (a resource) and then given the opportunity to give some to another child (an anonymous receiver) visiting the next day. Again, the child’s decisions are made in private and the anonymous recipient is not present, so social pressures should have less impact on their behavior, compared to if the recipient were present [11]. Overall, these studies reveal that even early in development, children are willing to share portions of their resources with anonymous others, even when not required to do so (e.g., [1,12]). Moreover, altruistic behaviors increase with age, such that a child’s propensity to donate any of their stickers to the recipient increases from the ages of 3–6 [1,12]. However, when children do donate, the number of stickers they donate does not appear to vary across this same age range, with 3–6 year olds sharing approximately the same amount, donating an average of 50% of non-preferred stickers but only 40% of higher-valued stickers [1].

This dictator paradigm, however, provides an opportunity to explore how children think about resource distribution. Although children will spontaneously share a portion of their resources with anonymous individuals, it is not clear how they determine how much of the resource to donate (or how much to keep). In the current study, we explored how sharing behavior is impacted by variations in the number of available resources and in the number of potential anonymous recipients in 3–11 year old children. In doing so, we were able to address three open questions regarding children’s spontaneous sharing behavior: (1) How do children decide how much of their windfall to distribute to others; that is, do they share in a proportional manner? (2) How much do children take into account the other recipient(s) when deciding how much to share; that is, in deciding how much to share, is the focus on how much is being shared, or on how much is being kept? and (3) Do these tendencies change with age?

#### Deciding how much to share: proportion versus absolute number

Previous developmental studies involving the dictator game have provided children with a limited, small number of resources (i.e., ten or fewer stickers; e.g., [1,4,12]). No studies to date have systematically varied the number of available resources to determine how this factor may impact children’s sharing behaviors. That is, do children give more if they have more to give? Generally speaking, in previous studies when children do share, they donate 3 or 4 (30%-40%) of the 10 preferred stickers allocated to them [1,13]. However, the significance of this number is unclear. Do children share in terms of proportions, such that they purposely give slightly less than half of their stickers (or alternatively, keep more than half)? Or is their focus on the absolute amount of stickers they donate, such that anonymous recipients are only deserving of 4 stickers, regardless of the number of available resources?

One possibility is that children decide to share a set *proportion* of their resources (e.g., 40%), such that the absolute number of resources shared increases proportionally with the total number of resources initially provided. Given a wealth of data indicating that children and adults represent number approximately in a proportional manner, such that numerical discriminations are ratio-dependent (i.e., obey Weber’s law; [14,15,16]), it would not be surprising if children shared resources in a proportional manner. In fact, work with adults using the DG support this account, revealing that adults are more likely to focus on proportion, not the absolute amount, when the number of available resources increases. Whether initially given $5 versus $10 [8] or $10 versus $100 [17,18] in a dictator game, the proportion of money adults shared with the other recipient is the same. Consistent with this account, children from higher SES environments have been found to share more in a DG setting than those from less affluent environments [13], suggesting greater access to resources may lessen the perceived value of each individual resource (sticker) in a proportional manner, resulting in children being more inclined to part with them. SES levels have also been found to correlate with a myriad of other social factors, including the quality of parent-child relationships, levels of violence, familial stress levels, parenting styles, and exposure to novel environments [19], all of which may also influence sharing behaviors. Thus, it is unclear whether these findings have any bearing on the question of how the number of available resources may influence sharing behaviors. Similarly, it has been found that when confronted with an individual who has no resources, preschoolers will ask a third party to help share resources with the indigent individual, but only if the child has fewer resources than the third party, suggesting that they perceive individuals with greater wealth as being more likely (and perhaps more responsible) to share their resources [20]. Thus, it may be the case that children spontaneously give a greater number of their resources when given a greater windfall of resources if their sharing behavior is dictated by proportional thinking.

On the other hand, developmental evidence suggests that young children may be more inclined to focus their attention on absolute number, especially in the context of sharing. In one study, 4–5 year-old children were asked to judge how nice two puppets were after each puppet had shared some of their resources with the child [21,22]. When both puppets shared the same proportion of their resources, but a different absolute number (Puppet A gave 2 of 4 resources, whereas Puppet B gave 6 of 12 resources), children were more likely to rate the puppet that gave the greater absolute amount (6 of 12) as being nicer. Moreover, when proportional giving was pitted against absolute giving (such that the puppet that gave the greatest absolute amount also gave a smaller proportion, i.e., Puppet A gave 3 of 4 whereas Puppet B gave 6 of 12), the children still preferred the puppet that gave the greater absolute amount, pointing to an early preference to focus on absolute number in prosocial contexts. The question then is whether this explicit preference for individuals who share a greater absolute number translates into children’s own sharing behaviors being dependent upon absolute number over proportionality. If so, then children may donate the same number of resources, regardless of the size of their windfall.

#### Generosity vs. fairness

When making sharing decisions, what is the child’s primary concern—is it a focus on how much of the resource they want to keep and how much they are willing to part with (i.e., how generous they are) or, alternatively, are they concerned with distributing resources in a fair manner? Put more explicitly, do children give more when there are more potential recipients?

The fact that children spontaneously share any of their resources when placed in a dictator game context indicates that they at least somewhat take into account the needs of others when distributing resources. Moreover, work involving the Ultimatum Game, in which children are given the option to accept or reject offers in a public forum in which resources (candy) are distributed between themselves and a social partner (another child), suggests an increasing sensitivity to fairness with age. Although children of all ages will typically reject offers in which they receive fewer resources than the social partner, it is not until approximately age 8 that children will similarly reject offers in which the child receives *more* resources than the social partner [23], indicative of a developing sense of fairness. If so, then children’s sharing decisions may be primarily based upon an overall preference towards equity amongst individuals, at least later in development. If children make sharing decisions based on *fairness*, then children should share a greater proportion of their resources when there are more recipients—that is, they should take into account the needs of others when determining how much to share.

On the other hand, reports reveal that children typically share less than half of their resources (~40%) in the standard DG [1,13], suggesting that they are less inclined to focus on fairness amongst recipients when distributing resources. Furthermore, evidence suggests that developmental trends towards a preference for equity (as found in e.g., [23]) may be driven by an attempt to appear fair in the eyes of others, such that children still continue to act selfishly when resource distribution decisions are made in private [5,12]. Thus, it may be the case that when presented with the option to share resources in private, children may simply ignore the number of recipients when making distribution decisions and instead egocentrically fixate on how much of the resource they plan to keep for themselves (regardless of contextual circumstances). If children focused on their *generosity* when allocating resources (e.g., [12,24]), as opposed to fairness, then they should donate the same amount of stickers, regardless of the number of potential recipients.

In sum, if the basis for sharing decisions is on how much of the resource they are going to keep/give away (i.e., how *generous* they are being), then children may not give any more of the resource when there are more recipients. Alternatively, if *fairness* considerations form the foundation for these decisions, then a greater proportion of resources should be shared when there are more potential recipients. Lastly, whether developmental differences exist in the dominance of these strategies (i.e., an early attention towards generosity, with a later emerging consideration for fairness?) is an open question.

### The present study

In the present study, 3- to 11-year-old children participated in an adapted version of the DG in which each child received either 12 or 30 stickers (i.e., they were “poor” or “rich” in stickers) and were given the opportunity to share with either one or two anonymous child recipients (all conditions administered between-subjects). These numbers were chosen so as to include values that were equally divisible by the total number of individuals involved (1 child + 1 or 2 recipients = 2 or 3 total individuals), while including one condition (12 stickers, 1 recipient) that approximately mirrored the 10-sticker condition of standard DGs (e.g., [1]). By doing so, we asked: Do the number of available resources and/or the number of potential recipients alter the likelihood of a child donating and/or the amount they donate? The answer to this question will speak to whether children’s giving is reflective of proportional or absolutist thinking about resources, as well as whether prosocial behavior is guided by a focus on generosity or fairness.

Additionally, the present study expands on previous research in an important way. By including a wide developmental age range, this study addresses how children’s prosocial sharing, and preference for fairness among others, develops past the age of seven. Previous studies have generally focused on children under the age of eight, thus it is unclear whether children’s allocations become more (or less) generous to the receivers beyond this age, and at what point they mimic those of adults.

## Methods

### Ethics statement

The Boston College Institutional Review Board in the Office for Research Protections (IRB protocol number 10.064) approved the ethics of this study. Informed, written consent was obtained from the parents or guardians of all of the children who participated in this study. Children under the age of 7 provided verbal assent and children over the age of 7 provided both verbal and written assent to take part in the research study.

### Participants

Four hundred and one children ages 3–11 years participated in this study. Participants were divided into four age groups: 3–4 year-olds (*N* = 111; *M* = 46.47 mos, *SD* = 7.1 mos; 61 females), 5–6 year-olds (*N* = 115; *M* = 69.0 mos, *SD* = 6.9 mos; 62 females), 7–8 year-olds (*N* = 106; *M* = 94.6 mos, *SD* = 7.0 mos; 62 females), and 9–11 year-olds (*N* = 69; *M* = 120.5 mos, *SD* = 9.9 mos; 36 females). Initial analyses indicated that 9, 10, and 11 year olds did not differ on any of our variables of interest (e.g., Proportion of children who donated: 9 yrs: 94.5% donated, 10 yrs: 100% donated, 11 yrs: 91.7% donated; *X* ^*2*^(2, *N* = 69) = 1.6, *p* = 0.446) thus the oldest age group included a three-year age range in order to maximize the size of this sample, allowing us to explore sharing behavior in an age group that has not been previously examined. Seventeen additional children were excluded from the study for failure to place all of the stickers in the available envelopes (*N* = 12), parental interference (*N* = 2), and not understanding/not following the rules of the game (*N* = 3). Although children who were excluded due to the last two factors varied unsystematically, the twelve children excluded for failing to place all of the stickers in available envelopes were all from the youngest age group (3 yrs: *N* = 8, 4yrs: *N* = 4). They did not vary consistently by condition, gender, or testing location (laboratory vs. museum). Children were recruited via phone or mail from local birth records for a one-time visit to our laboratory at Boston College in Chestnut Hill, MA, or from one of the two local museums, the Museum of Science, Boston, or the Boston Children’s Museum.

### Procedure

Children were pseudo-randomly assigned by age to one of four conditions in a 2 x 2 between-subjects design manipulating the number of available resources (12 or 30 stickers) and the number of anonymous recipients (1 or 2 recipients). Prior to completing the study, each child was asked to indicate which of four sticker types (sparkly heart, truck, smiley face, or star) was their favorite. The child’s preferred sticker type was then used for the DG task (modeled off of [1]).

In the standard condition, “12–1” (*N* = 98), the experimenter placed 12 of the child’s preferred stickers in front of them on a tray. Participants were told that all of the stickers were theirs to keep, but that another child would be visiting the next day and, if the participant wanted to, they could leave some stickers to be given to the other child. Importantly, the participant was told that this other child was just like them (i.e., same age and gender; [1]), thus making it unlikely that the participant would question whether the receiver would also value the sticker. Two envelopes were then placed on the tray on either side of the stickers, and the participant was told that one of the envelopes was for *their* stickers (i.e., the stickers the participant chose to keep), and the other envelope was for the stickers to be given to the other child (the anonymous receiver), if they chosen to share some. The placement of the envelopes was counter-balanced across participants. The experimenter verbally confirmed with the participant that they understood which envelope belonged to them and which would go to the receiver. Then, a privacy box [1] was placed over the tray so that the participant could make their sharing decision in privacy, and to ensure that sharing behavior was not indicative of a social signal to boost their reputation as a good person [5,25]. At the end of the game, participants were given the envelope containing the stickers they decided to keep, plus one additional sticker to thank them for participating. The number of stickers left in the other envelope for the receiver was recorded after the participant left the testing area and out of view of other potential participants.

In the “12–2” condition, (*N* = 105), the experimental procedure was identical; however, after being given 12 stickers, the participant was told that *two* anonymous children (of the same age and gender as the child) would be visiting the next day and were thus given 3 envelopes in which to distribute stickers (one for the participant and 2 for the recipients). To ensure that the participant did not confuse the envelopes, the two recipient envelopes were of the same color, but a different color from that of the participant’s envelope (e.g., two green envelopes and one red) and the experimenter again verbally confirmed with the participant that they understood which envelope belonged to them and which belonged to the children visiting the next day. In the “30–1” condition, (*N* = 106), participants were initially given 30 stickers to keep and/or distribute between themselves and one other recipient. Lastly, in the “30–2” condition, (*N* = 92), participants received 30 stickers to keep and/or distribute between themselves and two other recipients (thus they were given 3 envelopes).

## Results

### Propensity to Donate

**Fig 1:**
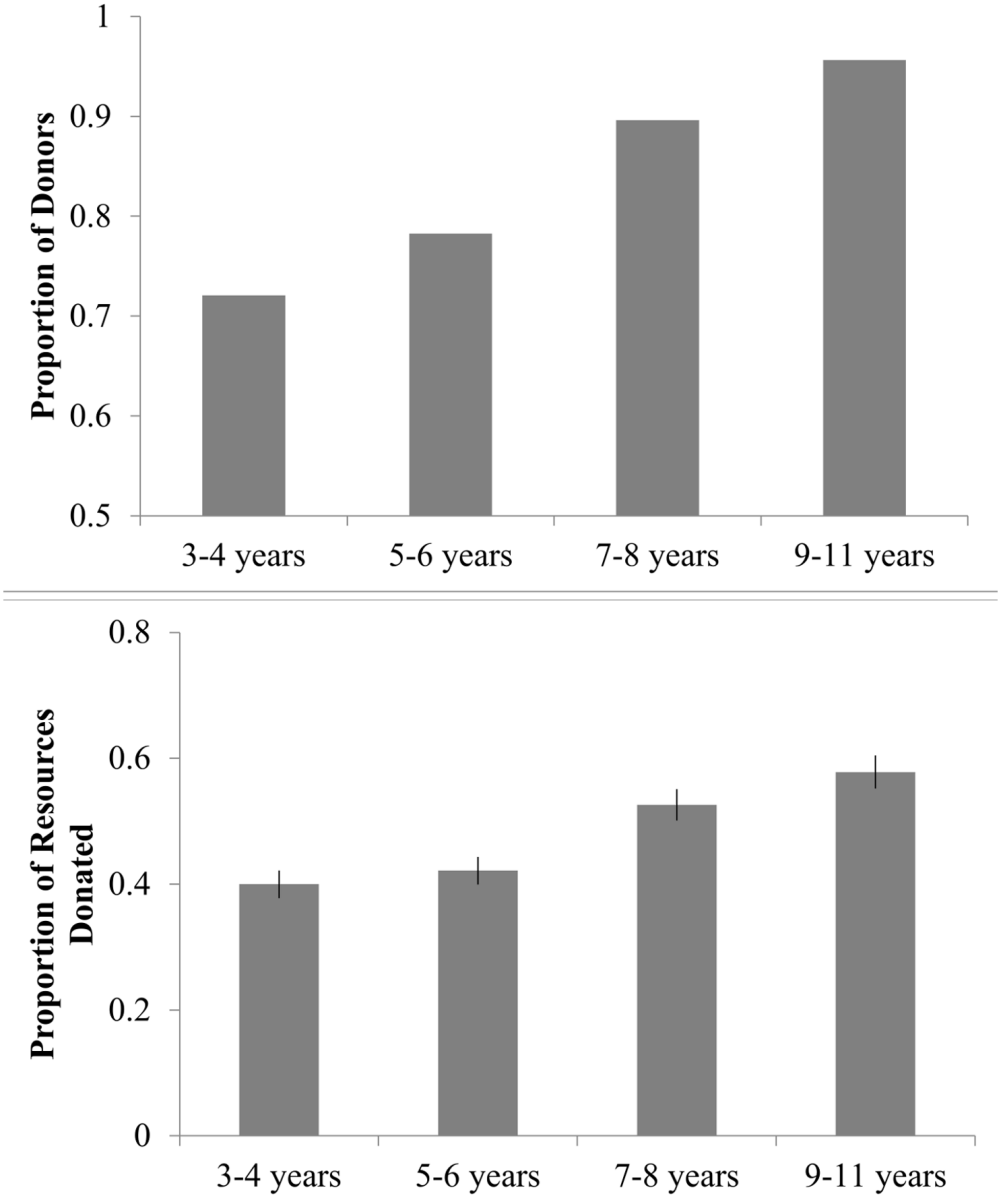
Increased Giving With Age. Children were more likely to donate resources to anonymous receivers with age (top). Moreover, of those children who donated their resources, older children donated a greater proportion of their resources on average than younger children (bottom). Error bars indicate standard error.

An overall chi-square analysis examining the number of children who chose to give any stickers (“givers” = 1) versus those who chose not to give any stickers (“non-givers” = 0) revealed that with age children were more likely to share than to not to share (*X* ^2^(3, *N* = 401) = 21.8, *p*<0.001; Fig 1). Further regression analyses confirmed that, although the probability of a child deciding to share increased with their age (in years; *Standardized Beta* = 0.230, *t*(400) = 4.7, *p*<0.001, 95% CI [0.048 to 0.117]), their decision was unaffected by both the number of resources available to the child and the number of recipients (Number of resources: *p*>.7, Number of recipients: *p*>.4; Proportion of givers: *M*_*12stickers*_ = 0.833; *M*_*30stickers*_ = 0.818; *M*_*1recipient*_ = 0.809; *M*_*2recipients*_ = 0.843). Similarly, gender did not significantly predict whether or not children chose to donate (85% of females donated vs. 80% of males; *p*>0.2; Model: *R* ^*2*^ = .059, *F*(4, 396) = 6.2, *p*<0.001).

### Donation Size

Initial analyses confirmed that the absolute number of resources shared differed significantly as a function of the number of resources initially given to the child (*F*(1, 299) = 258.6, *p*<0.001, *n* ^*2*^_*p*_ = 0.46), confirming that resource distribution decisions were not exclusively based upon a focus on the absolute number of resources donated (or kept). Thus, all further analyses were conducted on the proportion of resources shared to evaluate whether these decisions were proportion-based. Additionally, all subsequent analyses include only those children who decided to share any of their resources (“givers,” [1]).

**Fig 2:**
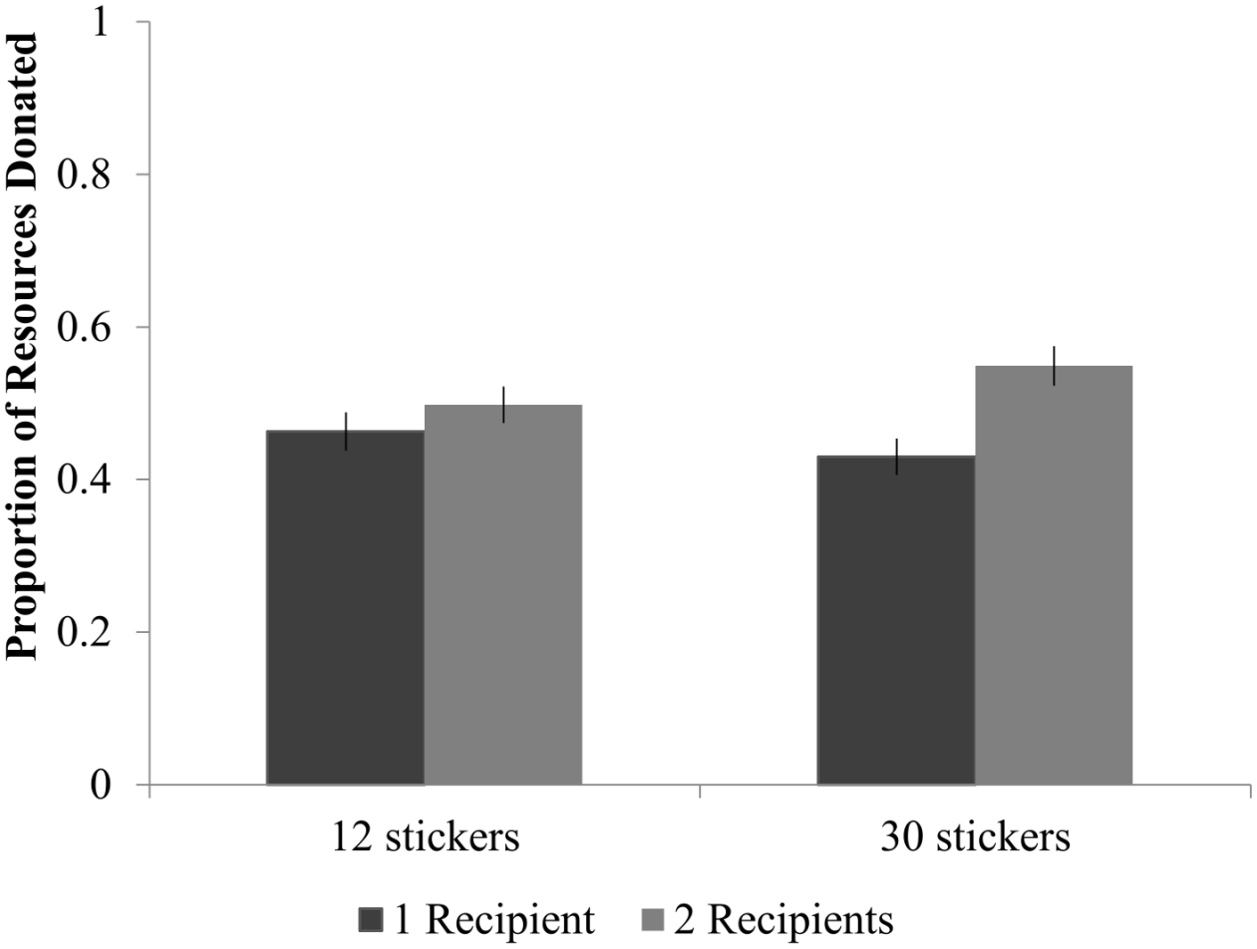
The frequency of children who donated the indicated number of stickers per age group in the 12-sticker (left panel) and 30-sticker (right panel) conditions.

A univariate ANOVA was conducted investigating the impact of the between-subjects factors of age (4: 3–4 years, 5–6 years, 7–8 years, 9–11 years), number of resources (2: 12 or 30 stickers), number of recipients (2: 1 or 2 anonymous recipients), and gender (2: female, male) on the *proportion* of resources shared. Results reveal that, in contrast to previous findings from studies with more limited age ranges [1], age significantly contributed to sharing behavior (*F*(3, 299) = 12.3, *p* < 0.001, $\eta_{p}^{2}$ = 0.110), such that children shared a greater proportion of their resources with age (Fig 1). In addition, the number of recipients significantly impacted children’s giving (*F*(1, 299) = 9.66, *p* = 0.002, $\eta_{p}^{2}$ = 0.031), such that children shared a greater proportion, on average, when presented with two recipients (*M* = 0.521) compared to when there was only a single recipient (*M* = 0.443). Moreover, the number of resources provided to the child did not singularly affect the proportion they shared (*F*(1, 299) = 0.125, *p* = 0.724, $\eta_{p}^{2}$ = 0.00), with children sharing a comparable proportion of resources when initially given 12 stickers (*M* = 0.478) as when they were given 30 stickers (*M* = 0.485), suggestive of a proportion-based strategy. This pattern did not vary as a function of age (age X number of resources interaction, *p* > 0.4) as might have been predicted by previous work on children’s views of others sharing behaviors [21]. Follow-up analyses confirmed that the *proportion* of stickers shared in the 12–1 and 30–1 conditions did not differ for any of the four age groups (all *p*’s>.18), nor did it differ for the 12–2 and 30–2 conditions in any age group (all *p*’s>.3, except 9–11 year olds *p* = .023, *NS with Bonferroni correction*). In contrast, when identical analyses compared the *absolute number* of stickers shared, significant differences were found between the 12–1 and 30–1 conditions and between the 12–2 and 30–2 conditions for each of the 4 age groups (all *p*’s≤0.001, all significant with *Bonferroni* correction). See Fig 2 and Table 1 for a breakdown of giving per age and condition.

**Table 1:** The mean proportion of resources shared (and standard error) in each of the four experimental conditions as a function of age group (means only include children who donated at least one sticker).

	12–1	30–1	12–2	30–2
**3–4 years**	.422 (.045)	.376 (.045)	.377 (.044)	.434 (.041)
**5–6 years**	.405 (.030)	.333 (.045)	.491 (.040)	.444 (.052)
**7–8 years**	.506 (.061)	.458 (.040)	.551 (.043)	.600 (.050)
**9–11 years**	.510 (.053)	.530 (.058)	.563 (.041)	.708 (.044)

**Fig 3:**
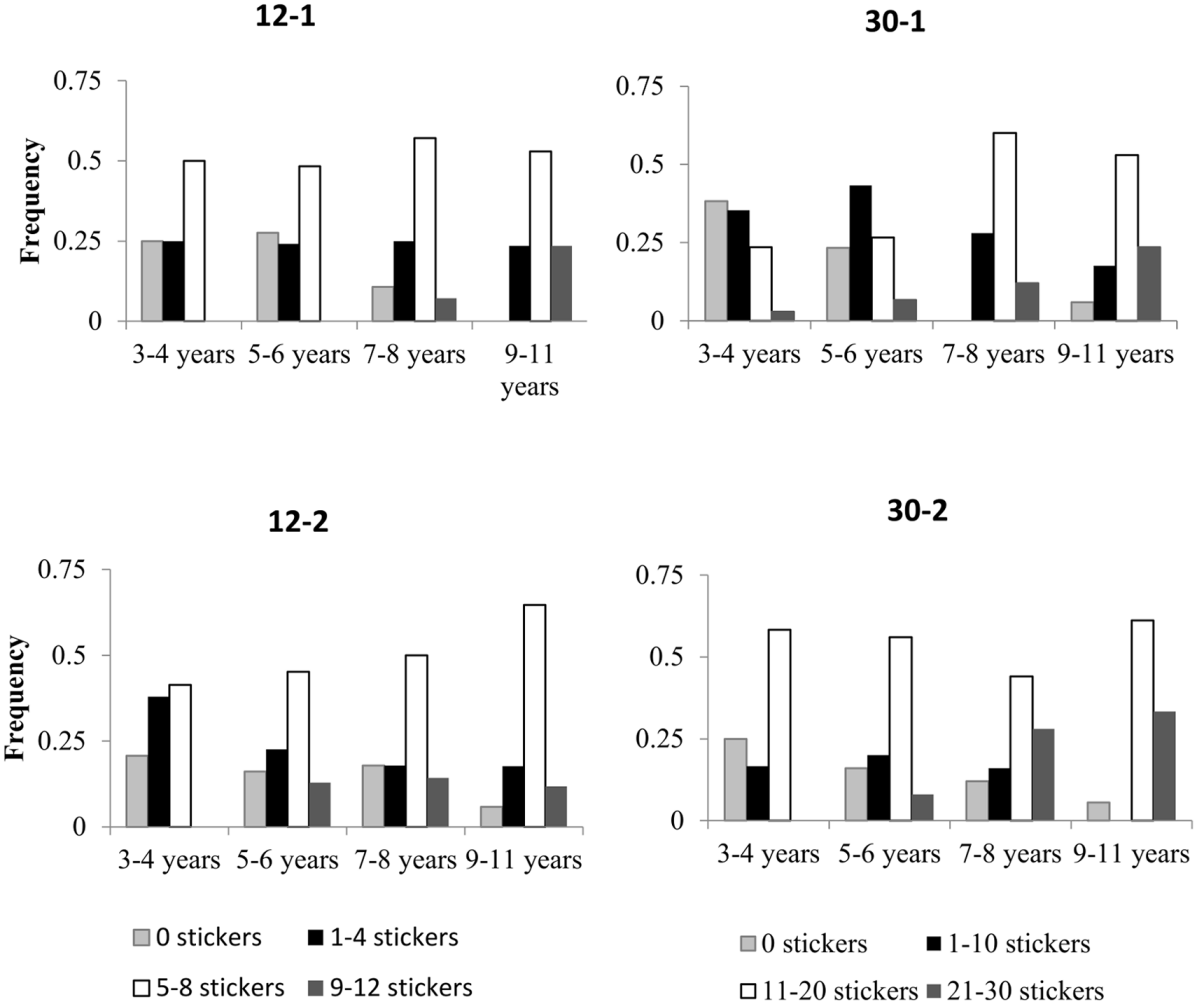
Sharing Across Conditions. Children donated a higher proportion of their stickers when there were multiple anonymous recipients, but only when they initially received 30 stickers. Error bars indicate standard error.

A marginal interaction between the number of resources and number of recipients (*F*(1, 299) = 2.87, *p* = 0.091, *approaching*, $\eta_{p}^{2}$ = 0.01) suggested that, although the number of initial resources given to the child did not impact the proportion of resources donated, children were slightly more sensitive to the number of recipients present when provided with a relative wealth of resources (Fig 3). That is, although a similar pattern was found in both cases, when children had relatively fewer stickers (12 stickers), their sharing behavior was less affected by the number of recipients (1 recipient: *M* = 0.461 vs. 2 recipients: *M* = 0.495; *t*(167) = 0.815, *p* = 0.416; *Cohen’s d* = 0.185) than when given 30 stickers initially (1 recipient: *M* = 0.424 vs. 2 recipients: *M* = 0.546; *t*(160) = 3.49, *p* = 0.001; *Cohen’s d* = 0.547).

**Fig 4:**
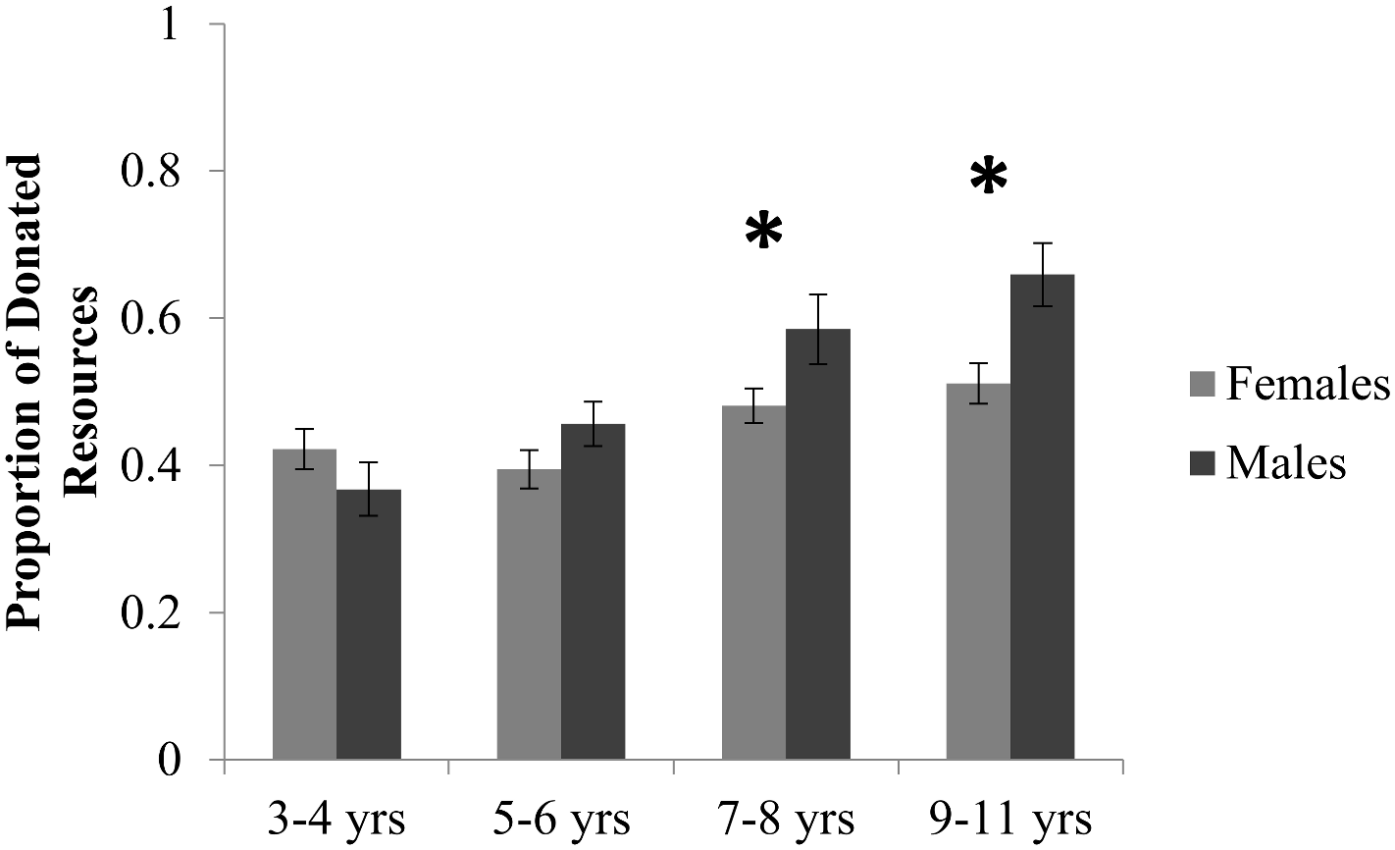
Distribution of Resources by Gender. While 3–6 year olds did not differ in the proportion of resources they shared by gender, 7–11 year old males donated a significantly greater proportion of their resources. Error bars indicate standard error.

Lastly, gender also impacted children’s donations. A main effect of gender (*F*(1, 299) = 6.67, *p* = 0.01, $\eta_{p}^{2}$ = 0.022; Fig 4) indicated that males (*M* = 0.517) donated a higher proportion of their resources than females (*M* = 0.453). A marginal interaction between age and gender (*F*(3, 299) = 2.53, *p* = 0.057, $\eta_{p}^{2}$ = 0.025) further indicated that it was the older children that drove this effect, such that the two youngest age groups shared proportionally similarly across gender (3–4 yrs: *M*_*Females*_ = 0.423 vs. *M*_*Males*_ = 0.368, *t*(78) = 1.2, *p*>0.2, *Cohen’s d* = 0.27; 5–6 yrs: *M*_*Females*_ = 0.398 vs. *M*_*Males*_ = 0.451, *t*(88) = 1.23, *p*>0.2, *Cohen’s d* = 0.26), whereas males in the oldest two age groups shared proportionally more of their resources than females (7–8 yrs: *M*_*Females*_ = 0.481 vs. *M*_*Males*_ = 0.586, *t*(93) = 2.11, *p* = 0.037, *Cohen’s d* = 0.44; 9–11 yrs: *M*_*Females*_ = 0.511 vs. *M*_*Males*_ = 0.659, *t*(64) = 3.0, *p* = 0.004, *Cohen’s d* = 0.75).

### Preference for equity amongst recipients

**Fig 5:**
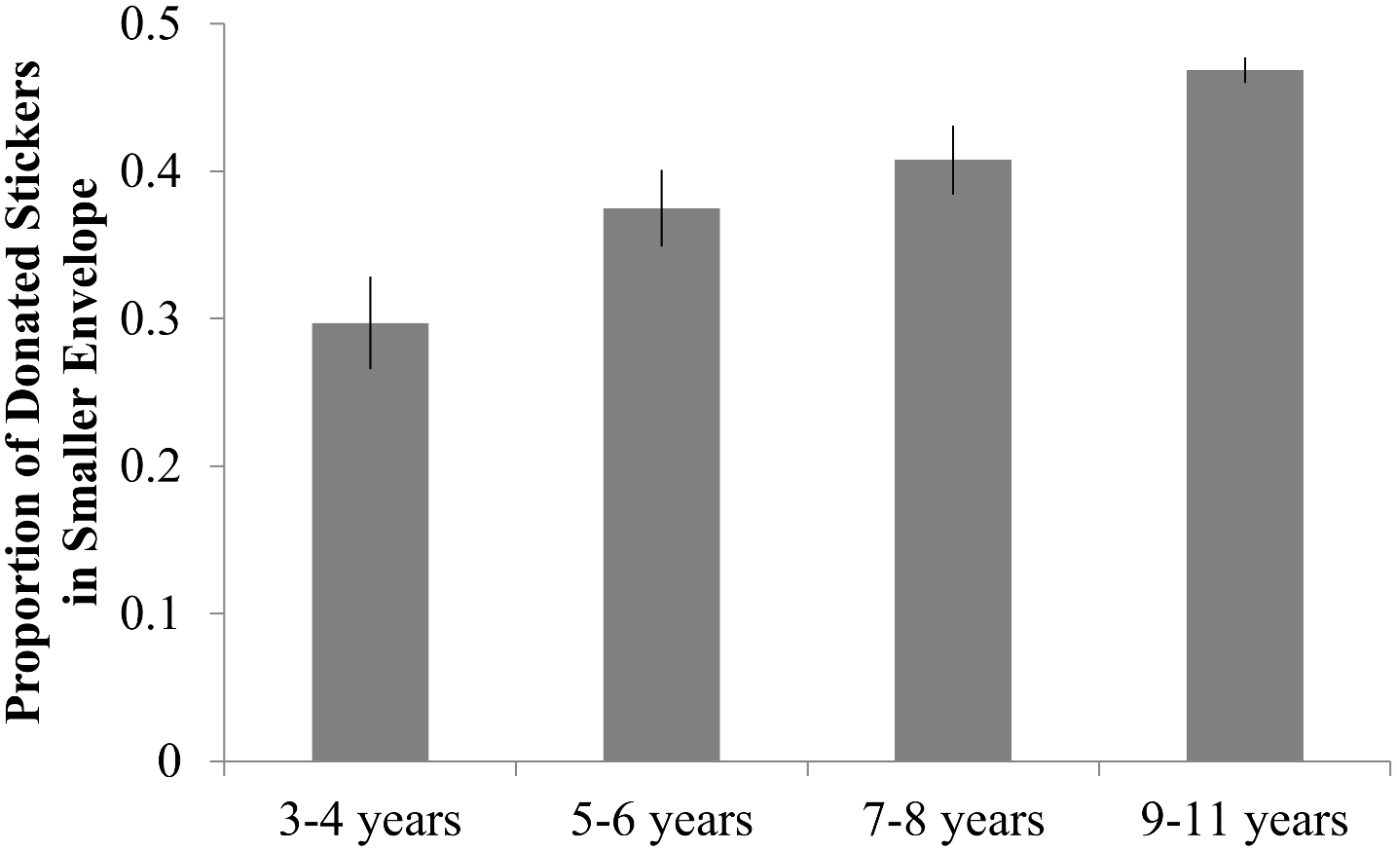
Preference for Equity Across Anonymous Receivers. In the multiple recipient conditions (12–2 and 30–2 combined), children became more sensitive to equity amongst the two anonymous receivers with age, with older children more likely than younger children to evenly distribute donated resources amongst the two envelopes. Error bars indicate standard error.

Additional analyses were conducted on data exclusively from the Two-Recipient conditions (12–2 and 30–2) to determine whether children took fairness into account when distributing resources across multiple recipients. That is, were children concerned with whether one anonymous recipient received more than another? To explore this question, a measure of equity preference was determined by computing the ratio of the number of stickers in the receiver’s envelope (i.e., the smaller envelope) containing the fewer stickers to the total number of stickers donated, such that a score of 0.5 indicates a perfect preference for equity, while a score of 0 indicates a perfectly uneven distribution of resources amongst the recipients. Of note, this measure is independent of how many stickers the child decided to share versus keep. That is, a child donating all of their stickers to the recipients could have the same preference for equity as a child donating only half of their stickers, so long as the relative distribution across the recipients’ envelopes was comparable. Importantly, children who shared only a single sticker were excluded from this analysis because it was impossible for them to distribute a single resource equally amongst two recipients. For example, if a child donated 4 stickers to one recipient and 12 stickers to another recipient, the child’s equity preference would be computed at 4/(4+12) = 0.25. Using this measure, regression analyses entering number of resources (2: 12 or 30; coded as: 1, 2), age group (4: 3–4 years, 5–6 years, 7–8 years, 9–11 years; coded as: 1, 2, 3, 4), and gender (2: female, male; coded as: 1, 2, respectively) as predictors of equity preference scores revealed that neither the number of available resources nor the participant’s gender influenced children’s preferences for equity (Number of Resources: *Standardized Beta* = 0.01, *t*(160) = 0.128, *p* = 0.898, 95% CI [-0.047 to 0.054]; Gender: *Standardized Beta* = -0.049, *t*(160) = 0.651, *p* = .516, 95% CI [-0.067 to 0.034]). The probability of equal sharing did increase as a function of age group though (*Standardized Beta* = 0.336, *t*(160) = 4.50, *p*<0.001, 95% CI [0.030, 0.077]; Model *R* ^*2*^ = .115; *F*(3, 160) = 6.9, *p*<0.001; Fig 5), such that older children were more likely distribute resources evenly across the envelopes for the two recipients than younger children.

## Discussion

Results of this study shed light on how children share resources with anonymous recipients when the number of resources and recipients varies. Importantly, results replicated a previous findings revealing that children spontaneously engage in altruistic sharing when presented with a windfall, even when not required to do so, with over 80% of children in the current study choosing to share at least some of their stickers with (an) unknown recipient(s). Moreover, data reveal that age was positively correlated with the likelihood of sharing, consistent with previous findings (e.g., [1]).

However, importantly, the current study extends previous work by exploring how the number of available resources and the number of potential recipients altered children’s giving. Our results reveal that neither the number of stickers available to the child nor the number of potential recipients impacted the likelihood of a child choosing to donate any of their stickers. In contrast, for those children who did share, the proportion of resources that they shared did vary as a function of the number of recipients (coupled with the number of stickers that they initially received) and their age. Unsurprisingly, children did donate a greater absolute number of resources when their initial windfall was greater, but the actual proportion of resources they shared did not vary as a function of the number of available resources. Together, these findings suggest that the decision *whether* to share may be more impervious to contextual circumstances than the decision of *how much* to share. Thus, whether a child chooses to share likely reflects a stable individual trait requiring intrinsic motivation, whereas how much a child shares may instead be extrinsically motivated by the circumstances.

### Developmental Changes

Although previous studies did not find sharing behavior among donors to vary with age [1], our data did reveal age-related differences (with a medium effect size), with older children sharing a greater proportion of their resources than younger children. Importantly, in contrast to previous studies, participants in our study extended into middle childhood, and it appears that age-related differences in this measure did not emerge until sometime after age six, suggesting that children’s sensitivity to the needs or wants of others may continue to mature or change over childhood and with experience.

Other than increased giving, age-related differences were not observed in terms of the pattern of sharing across conditions. That is, our analyses did not reveal any interactions involving age and our variables of interest (number of resources or number of recipients), suggesting that these variables affected sharing similarly across childhood. Follow-up analyses performed on the data from each age group confirmed that the proportion of resources donated did not differ as a function of the size of the initial windfall in either the 1-envelope or 2-envelope conditions across our age groups. In contrast, comparable analyses revealed that the absolute number of resources donated did differ in the 1-envelope and 2-envelope conditions across all age groups, consistent with a proportion-based account.

However, additional analyses on resource distribution in the 2-recipient condition did reveal developmental trends in the likelihood that resources were distributed evenly amongst multiple recipients. Whereas older children were careful to ensure fairness in the distribution of resources amongst anonymous donors—dividing donated stickers across multiple recipients with an attention towards equity—younger children did not take into account equity amongst recipients, randomly allocating stickers in the 2-recipient conditions.

If we assume that children first made the decision of how much of their resource they would share (and how much they would keep for themselves) and then, after this, decided upon the allocation of their donation to the two different envelopes, then this aspect of the study should be considered a third-party sharing task. As such, these findings are in contrast to other studies revealing that even infants (as young as 19 months of age; [26]) are sensitive to equal resource distribution amongst two recipients in third-party tasks. However, it should be pointed out that a number of design features made this task distinct from other third-party tasks, which may have contributed to the different pattern of findings. For example, the complexity of the task demands (a multi-step combination of a first-party task (distributing resources between oneself and others) and third-party task (then distributing resources amongst two recipients), coupled with the large number of resources distributed (12 or 30), may have been too cognitively taxing for the young children, making them less likely to attend to equity amongst third parties. Moreover, it is not clear whether (as assumed) children made their donation decisions in sequence. That is, children may have simultaneously negotiated how much to keep for themselves along with how to allocate stickers across the two recipients, making the cognitive constraints of the first-party decisions impact their ability to make third-party decisions. Furthermore, although the envelope for the participant’s resources differed in color from that of the recipients, the envelopes for the two recipients were the same color, making it more difficult for children to keep track of which envelope they may have filled previously. Regardless of the reason that the youngest children did not generally distribute stickers evenly amongst recipients, even 3–4 year olds took others into account by donating more when there were more recipients when their initial windfall was large (30 stickers), suggesting that young children are sensitive to the needs of others inasmuch as it pertains to themselves—they give more of their own resources when there are more individuals in need (but only when they have enough of the resource to share), but they may not consider fairness in the distribution of these resources among other individuals (when they are not part of the equation) until later in childhood (around 5–6 years of age).

### Proportion versus Absolute Number

Analyses of how much children shared when either relatively poor or rich in stickers revealed that children allocated resources in a fairly proportional manner, allocating a different absolute number of resources, but a similar proportion, regardless of the number of initial resources available. These findings were confirmed both at the group level, and when analyzing average donations as a function of age group within the 1-envelope and 2-envelope conditions separately. While it should be noted that non-significant findings can neither prove nor disprove a proportion-based account of children’s sharing in our task, however significant results from analyses of the absolute number of stickers donated do rule out the possibility that children fixated upon the absolute number of stickers when making their donation decisions. This finding contrasts with previous work with preschoolers revealing an early preference for individuals who share a greater absolute amount of resources, regardless of how many resources the individual has [21]. Thus, findings imply dissociations between children’s explicit preferences in a third-party sharing context (as measured in [21]) and their sharing behaviors within a first-party context, mirroring reported differences between children’s explicit judgments of how much someone *should* share versus how much they *actually* choose to share [12]. When presented with an explicit comparison, children’s preferences may appear somewhat immature, focusing on absolute amount. In contrast, when children are placed in a circumstance in which they must make the decision of how much of a resource to distribute, their behavior does not appear to differ from that of adults [8,17,18], with behavior consistent with a reliance upon the proportion of resource they are willing to allocate to others (consistent with the ratio-dependence observed in their numerical representations; e.g., [14]).

Why is it that children did not give based upon absolute number despite judging others based upon the absolute number of resources that they give [21,22]? One possibility may be that the context in which sharing decisions were made in the current study promoted intuitive thinking about proportions by prompting children to think about the initial total quantity available (i.e., the denominator in the ratio). It should be noted that we are not suggesting that children made explicit proportion computations (e.g., 40% of 12 or 30) when deciding how much to donate; instead, we argue that children’s proportional reasoning was *intuitive*, such that they may have used a proportion-based heuristic (e.g., “slightly less than half”) to determine how many stickers to donate. There is ample evidence to support the claim that the ability to intuitively process proportional information is present as early as infancy [27–33]. However, while children reveal that while they are similarly capable of understanding proportional information, when this information is presented in a discrete format (i.e., numerical), then children as old as 6-years of age show a greater reliance upon absolute number [34,35,36]. Importantly, proportional reasoning requires consideration of a ratio of the total number of resources shared to the total number of resources available. A greater focus on absolute number can reflect a disregard for the denominator in this equation—an error that children have demonstrated time and time again in proportional judgments tasks [34,36] and that even adults are prone to when dealing with fractions [37,38]. When children are trained to attend to the denominator, however, their judgments become much less reliant upon absolute number [35]. By distributing a windfall of resources initially to participants, we may have made the total number available more salient to the children, highlighting the denominator in the proportional equation, thereby making proportional reasoning more intuitive for the children. Ng and colleagues [22] found that when children are asked to judge the niceness of individuals sharing resources, children prefer others who share a greater proportion of resources (over those who share a greater absolute number) when the particular context promoted intuitive thinking about equity amongst participants. Our data suggest that contexts that highlight the total number of available resources (i.e., the denominator) may similarly engage proportional reasoning in young children. Future work may explore whether similar strategies of highlighting the initial amount available may also make proportional reasoning more intuitive in the case of mathematical instruction.

### Generosity versus Fairness

Additionally, data reveal that children modified how much they donated based upon the number of potential recipients, donating more when there were more anonymous recipients. Although the effect size was small, children shared approximately 40% of their preferred stickers when there was a single recipient [1,13], and shared significantly (though not proportionally) more when there were two recipients (*M* = 48.9%). These findings are primarily consistent with a fairness-based account of children’s sharing behavior; that is, children donated more stickers when faced with multiple recipients in order to maximize equity amongst the recipients. Thus, it is not the case that children were fixated on exactly how much of the resource they wanted to keep for themselves and/or how much they were willing to give away, but instead, children varied the size of their donations based upon the particular circumstances presented to them in an effort to distribute resources (somewhat) fairly across recipients.

Notably, however, a marginal interaction highlighted the fact that this increased sharing with more recipients was only significant in conditions in which children were initially given a large windfall of resources (30 stickers). Although children who were given a relatively small windfall (12-stickers) did donate more stickers when confronted with 2 potential recipients (compared to 1-recipient), this difference was not statistically different. In contrast, children who had a relatively large windfall (30 stickers) donated a statistically greater number of stickers when there were more recipients. Thus, although overall children appeared concerned with fairness, it was only when children were “sticker rich” that they were inclined to take into account the number of potential recipients when deciding how much of their resource to distribute.

Importantly, although children generally showed a preference for equity, their distributions were not exactly fair. Across all four conditions, children still shared less than an equal distribution between themselves and the recipient(s), allocating the greatest proportion of stickers for themselves. For example, in the most generous 30–2 condition, children (on average) donated more than half of their resources (~54%), to anonymous recipients. Even so, this generous donation still equated to a greater proportion of resources for themselves (~46%) with smaller amounts for each of the two recipients (27%). Thus, even though children appeared sensitive to the proportion of stickers to share and the number of potential recipients when sharing resources, they still kept more for themselves than would be dictated by an equitable distribution.

Notably, however, the oldest participants (9–11 year olds) did not appear to fall into this category. In contrast to younger participants, the oldest children were generally inclined to share equally amongst all individuals involved in the dictator game, donating (on average) 50% of their resources when there was one recipient, and 66% of their resources when there were two recipients. While this pattern may have been (at least partially) driven by a decreased interest in stickers at this later age, findings are also consistent with literature suggesting that a preference for equity may not emerge until middle childhood [12,23]. Although some studies have reported equal sharing as early as 6 years of age [12], these studies have required children to make their donations in view of the experimenter. Evidence suggests that children are highly aware of the appearance of fairness [5,12,39,40], such that they attempt to appear fair as a signal of their impartiality to others. Thus, it is not surprising that we find a later emergence of equal giving in our subjects when decisions were made in the context of a privacy box, which made social signaling irrelevant. Thus, our data suggest that in the context of apparent privacy, children continue to unequally distribute resources between themselves and anonymous receivers well into middle childhood.

### Gender Differences

Analyses also revealed an unexpected main effect of gender (and a marginal age X gender interaction) revealing that males (in particular, older males) donated more than females in our task. It should be noted that the effect sizes of both of these effects were quite small, though. Although not initially part of the research questions presented, these results contrast with previous findings of either no gender difference, or even a slight female bias in resource distribution settings (e.g., [13, 41,42,43]; for a review, also see: [44]). While it is unclear why we found a (small but significant) male bias in resource distribution in our study, we speculate that this may have been driven by (unintentional) differences in subjective value of the stickers donated. Although participants were always given their preferred sticker (of four alternatives) to distribute, it may be the case that males overall valued these stickers less than their female counterparts, leading to a greater proportion of sharing. Although we chose an assortment of sticker choices to be equally valued by boys and girls in our study, it is possible that boys found our stickers to be less appealing than the girls did. Relatedly, since gender differences emerged with age, it is also possible that older boys value stickers less than girls of the same age. Blake and Rand [1] reported that low-value stickers were donated at a significantly higher rate than high-value stickers. If it is the case that boys valued our sticker choices less (and/or that older boys generally value stickers less than older girls), then this may account for why they donated stickers at a higher rate than their female counterparts. More research is needed, however, to determine whether the males did value the stickers less and/or whether similar higher rates of donations are observed in male children in other prosocial games involving different resources (e.g., candy). Moreover, the possibility that previously reported gender differences in altruistic behavior (revealing that females donate at a higher rate than males) are driven (at least partially) by differences in the subjective value of distributed resources should be considered.

### Limitations

The present study contained several limitations that should be considered. First, the cross-sectional design of this study leaves open questions relating more specifically to mathematical understanding. Although the current study specifically sought to investigate whether children think about resource distribution proportionally versus in an absolute manner, manipulating the number of stickers initially given to the participant in a between-subject fashion limits the claims that can be made about children’s proportional reasoning abilities. Second, while previous work has used stickers as a resource in DG contexts, it is unclear whether depreciating value of this resource with child’s age and/or with greater number of resources may have unduly influenced results. Future work should explore whether a similar pattern of results may be obtained for a much more highly valued resource (e.g., candy). Third, although this particular design has been successfully used with children in the past, older participants may have been less likely to believe the manipulations. For example, they may have been less likely to believe that “another child, just like you, will be visiting here tomorrow” and/or less likely to believe that their sharing decisions would be completely private (despite the privacy box). In fact, according to Experimenter Notes, on at least a single occasion, a child volunteered at the end of the study “There isn’t *really* another little girl coming here tomorrow, is there?” To verify that our findings with older children truly reflect prosocial tendencies in this age group, converging lines of evidence derived from distinct paradigms should be obtained.

### Conclusion

In conclusion, results extend the developmental timeline established in previous work with children and suggest that older children mimic the prosocial behavior seen in adulthood, both in terms of their propensity to share valued resources and their willingness to increase the amount of resource they allocate to others based on either an increase in the number of recipients or an increase in the resources available. Together, results of the current study replicate previous findings revealing that young children readily engage in altruistic sharing, and extend this work to reveal that how much a child gives (but not whether the child gives or not) varies depending upon the context (the number of available resources and the number of receivers). A breadth of research on prosocial tendencies across development suggests that different forms of prosocial behavior may be derived from different mechanisms and ecological foundations [45]. The evolution of resource distribution is particularly interesting, in light of the fact that appearing impartial or fair (e.g., [24,39, 46,47] is not always a good strategy or outcome for the individual making that decision. However, even young children who do not formally engage in these practices (e.g., in school, with others) show a preference for fairness and equity, as well as a preference for individuals who similarly value these behaviors (e.g., [40]). In the present study, we demonstrate: (1) Children across development allocate resources consistent with a proportional—rather than absolute—manner, as adults do; and (2) Children seem to consider fairness, rather than their own generosity per se, when allocating resources.

## References

[1] Blake P. and Rand D. (2010). Currency Value Moderates Equity Preference among Young Children. Evolution and Human Behavior 31, 210–18.

[2] Hamlin J.K., & Wynn K. (2011). Five- and 9-month-old infants prefer prosocial to antisocial others. Cognitive Development, 26, 30–39. 21499550 10.1016/j.cogdev.2010.09.001 PMC3076932

[3] Hamlin J. K., Wynn K., Bloom P., & Mahajan N., (2011). How infants and toddlers react to antisocial others. Proceedings of the National Academy of Sciences. 108, 19931–19936. 10.1073/pnas.1110306108 PMC3250174 22123953

[4] Fehr E., Bernhard H., and Rockenbach B. (2008) Egalitarianism in Young Children. Nature 454(7208), 1079–083. 10.1038/nature07155 18756249

[5] Shaw A., Montinari N., Piovesan M., Olson K. R., Gino F., & Norton M. I. (2013). Children develop a veil of fairness. Journal of Experimental Psychology: General. 10.1037/a0031247 23317084

[6] Bolton G. & Ockenfels A. (2000). A theory of equity, reciprocity and competition. American Economic Review, 90, 166–193.

[7] Fehr E. & Fischbacher U. (2003). The nature of human altruism. Nature, 425, 785–791. 14574401 10.1038/nature02043

[8] Forsythe R., Horowitz J., Savin N., and Sefton M. (1994). Fairness in Simple Bargaining Experiments. Games and Economic Behavior 6, 347–69.

[9] Hsee C. K., Zhang J., Lu Z. Y., & Xu F. (2013). Unit asking: A method to boost donations and beyond. Psychological Science, 24(8), 10.1177/0956797613482947 23907547

[10] Camerer C. (2003). Behavioral Game Theory: Experiments in Strategic Interaction. Princeton University Press.

[11] McAuliffe K., Blake P.R., Kim G., Wrangham R.W., & Warneken F. (2013). Social influence of inequity aversion in children. PLoS One, 8(12), e80966 10.1371/journal.pone.0080966 24312509 PMC3846671

[12] Smith C. E., Blake P. R., & Harris P. L. (2013). I should but I won’t: Why young children endorse norms of fair sharing but do not follow them. PLoS ONE 8(3), e59510–e. 10.1371/journal.pone.0059510 23527210 PMC3603928

[13] Benenson J., Pascoe J., and Radmore N. (2007). Children's Altruistic Behavior in the Dictator Game. Evolution and Human Behavior 28(3), 168–75.

[14] Jordan K. E., & Brannon E. M. (2006). A common representational system governed by Weber’s law: Nonverbal numerical similarity judgments in 6-year-olds and rhesus macaques. Journal of Experimental Child Psychology, 95(3), 215–229. 16808924 10.1016/j.jecp.2006.05.004

[15] Barth H., LaMont K., Lipton J., Dehaene S., Kanwisher N., & Spelke E. S. (2006). Non-symbolic arithmetic in adults and young children. Cognition, 98, 199–222. 15876429 10.1016/j.cognition.2004.09.011

[16] Droit-Volet S., Clement A., Fayol M. (2007). Time, number and length: similarities and differences in discrimination in adults and children. Q. J. Exp. Psychology, 61, 1827–1846. 10.1080/17470210701743643 19031154

[17] Carpenter J., Verhoogen E., and Burks S. (2005). The Effect of Stakes in Distribution Experiments. Economics Letters 86(3), 393–98.

[18] List J. and Cherry T. (2008). Examining the Role of Fairness in High Stakes Allocation Decisions. Journal of Economic Behavior & Organization 65(1), 1–8.

[19] Evans G. W. (2004). The Environment of Childhood Poverty. American Psychologist. Feb-Mar, 59(2), 77–92. 14992634 10.1037/0003-066X.59.2.77

[20] Paulus M., Gillis S., Li J., & Moore C. (2013). Preschool children involve a third party in a dyadic sharing situation based on fairness. Journal of Experimental Child Psychology, 116, 78–85. 10.1016/j.jecp.2012.12.014 23597498

[21] McCrink K., Bloom P., and Santos L. (2010). Children's and Adults' Judgments of Equitable Resource Distributions. Developmental Science 13(1), 37–45. 10.1111/j.1467-7687.2009.00859.x 20121861

[22] Ng R., Heyman G.D., & Barner D. (2011). Collaboration promotes reasoning about resource distribution in young children. Developmental Psychology, 47(5), 1230–1238. 10.1037/a0024923 21806299 PMC3168685

[23] Blake P.R., & McAuliffe K. (2011). I had so much it didn’t seem fair: Eight-year-olds reject two forms of inequity. Cognition, 120(2), 215–224. 10.1016/j.cognition.2011.04.006 21616483

[24] Shaw A., & Olson K.R. (2012). Children discard a resource to avoid inequity. Journal of Experimental Psychology: General, 141(2), 382–395. 22004168 10.1037/a0025907

[25] Nowak M. (2006). Five rules for the evolution of cooperation. Science, 314, 1560–1563. 17158317 10.1126/science.1133755 PMC3279745

[26] Sloan S., Baillergeon R., & Premack D. (2012). Do infants have a sense of fairness? Psychological Science, 23(2), 196–204. 10.1177/0956797611422072 22258431 PMC3357325

[27] McCrink K., & Wynn K. (2007). Ratio abstraction by 6-month-old infants. Psychological Science, 18(8), 740–745. 17680947 10.1111/j.1467-9280.2007.01969.x

[28] Xu F., & Garcia V. (2008). Intuitive statistics by 8-month-old infants. Proceedings of the National Academy of Sciences of the United States of America, 105, 5012–5015. 10.1073/pnas.0704450105 18378901 PMC2278207

[29] Offenbach S.I., & Gruen G.E. (1984). Development of proportional response strategies. Child Development, 55.3, 963–972. 6734330

[30] Spinillo A.G., & Bryant P. (1991). Children’s proportional judgments: The importance of “half”. Child Development, 62.3, 427–440.

[31] Xin Z., & Han Y. (2014). The developmental and interventional research on lower graders’ concept of equivalent fraction. Acta Psychologica Sinica, 46.6, 791–806.

[32] Xu F., & Denison S. (2009). Statistical interference and sensitivity to sampling in 11-month-old infants. Cognition, 112, 97–104. 10.1016/j.cognition.2009.04.006 19435629

[33] Ziqiang X., & Guofang L. (2011). The development of children’s non-symbolic calculation ability of whole-number and fraction and its relationship with number memory. Psychological Science (China), 34.3, 520–526.

[34] Boyer T.W., Levine S.C., & Huttenlocher J. (2008). Development of proportional reasoning: Where young children go wrong. Developmental Psychology, 44(5), 1478–1490. 10.1037/a0013110 18793078 PMC2597581

[35] Hurst M. & Cordes S. (2015). Intuitive proportional reasoning in children and adults. Manuscript in preparation.

[36] Jeong Y., Levince S.C., & Huttenlocher J. (2007). The development of proportional reasoning: Effect of continuous versus discrete quantities. Journal of Cognition and Development, 8:2, 237–256.

[37] Hurst M. & Cordes S. (2015). Comparing fractions, decimals, and whole numbers: Rational number understanding in adults. Journal of Experimental Psychology: Human Perception and Performance. 10.1037/xhp0000140 26389612

[38] Reyna V.F., & Brainerd C.J. (2008). Numeracy, ratio bias, and denominator neglect in judgments of risk and probability. Learning and Individual Differences, 18(1), 89–107.

[39] Shaw A. (2013). Beyond “to Share or Not to Share”: The impartiality account of fairness. Current Directions in Psychological Science, 22, 413–417.

[40] Shaw A., DeScioli P., & Olson K.R. (2012). Fairness versus favoritism in children. Evolution and Human Behavior, 33, 736–745.

[41] Gummerum M., Takezawa M., & Keller M. (2009). The influence of social category and reciprocity on adults’ and children’s altruistic behavior. Evolutionary Psychology, 7(2), 295–316.

[42] Ongley S.F., Nola M., & Malti T. (2011). Children’s giving: Moral reasoning and moral emotions in the development of donation behaviors. Frontiers in Psychology, 5, 458. 10.3389/fpsyg.2014.00458 PMC4033050 24904474

[43] Stewart S.M., & McBride-Chang C. (2000). Influence of children’s sharing in a multicultural setting. Journal of Cross-Cultural Psychology, 31, 333–348.

[44] Eckel C., & Grossman P. (2008). “Differences in the economic decisions of men and women: Experimental Evidence”. Handbook of Experimental Economics Results, Elsevier, 1(4), 509–519.

[45] Paulus M. (2014). The emergence of prosocial behavior: Why do infants and toddlers help, comfort, and share? Child Development Perspectives, 8(2), 77–81.

[46] Shaw A., & Olson K.R. (2013). All inequity is not equal: Children correct inequalities using resource value. Frontiers in Psychology, 4, 393 10.3389/fpsyg.2013.00393 23882227 PMC3715726

[47] Shaw A., & Olson K.R. (2014). Fairness as partiality aversion: The development of procedural justice. Journal of Experimental Child Psychology, 119, 40–53. 10.1016/j.jecp.2013.10.007 24291349

